# Profiling and Mapping Quality Improvement and Patient Safety Research Frameworks: Protocol for a Scoping Review to Guide Integration

**DOI:** 10.12688/hrbopenres.14173.3

**Published:** 2026-04-28

**Authors:** Siobhán E. McCarthy, Jill Poots, John McCaffrey, Emily O'Dowd, Anna V. Chatzi, Killian Walsh, Jennifer A. Pallin, Clíodhna McGowan, Tommy Kyaw Tun, John Brennan, Samuel Cromie, Edward W. Gregg

**Affiliations:** 1 Graduate School of Healthcare Management, RCSI University of Medicine and Health Sciences, Dublin, Ireland; 2 Centre for Innovative Human Systems, School of Psychology, Trinity College Dublin, Dublin, Ireland; 3 School of Population Health, RCSI University of Medicine and Health Sciences, Dublin, Ireland; 4 Department of Surgical Affairs, RCSI University of Medicine and Health Sciences, Dublin, Ireland; 5 School of Nursing and Midwifery, University of Limerick, Limerick, Ireland; 6 RCSI Library, RCSI University of Medicine and Health Sciences, Dublin, Ireland; 7 School of Medicine, RCSI University of Medicine and Health Sciences, Dublin, Ireland; 8 Royal College of Physicians of Ireland, Dublin, Ireland; 9 School of Public Health, Imperial College London, London, UK

**Keywords:** quality improvement, patient safety, research priorities, co-design, conceptual framework, scoping review

## Abstract

A core remit of the Evidence Based QUality Improvement and Patient Safety (EQUIPS) Research Network (2024–2029) is to inform research priorities for the Irish healthcare system. Previously, research prioritisation exercises have treated quality improvement (QI) and patient safety research as separate fields. Given the growth of QI research, an integrated approach is required to generate a full view of the continuum of knowledge generation and translation activities in quality and safety contexts. The co-creation of a Conceptual Framework for Quality Improvement and Patient Safety Research among researchers, knowledge users and patient groups is required to outline important topics, research questions and methodologies, applicable to all areas of healthcare. A scoping review of the literature is first needed to examine existing QI and patient safety research frameworks to inform the development of a harmonised conceptual framework for the Irish context. Methods The scoping review will follow JBI guidelines for developing and co-creating scoping review protocols with knowledge users. The completed review will be reported according to the PRISMA extension for Scoping Reviews, and Guidance for Reporting Involvement of Patients and the Public 2 (GRIPP2) checklist which supports enhanced reporting of patient and public involvement in research. We will search the following databases from 2000–2025 focussing on literature sources from developed countries: Ovid MEDLINE, Embase, CINAHL, Scopus, Cochrane Library, Web of Science. We will map included papers using an extraction template developed to fulfil the study aims. Two reviewers will independently screen studies, and discrepancies will be addressed by consensus using a third reviewer. Dissemination Findings will be used to inform the development of a meta-framework that will be used to guide QI and patient safety research priority setting, measurement and monitoring, implementation, and knowledge translation activities in Ireland, at local and national levels. OSF https://doi.org/10.17605/OSF.IO/FGWUA

## Introduction

A core aim of the Evidence Based QUality Improvement and Patient Safety (EQUIPS) Research Network (2024–2029), a collaboration of Irish Universities, patient representatives, and the Health Service Executive (HSE) in Ireland, is to inform research priorities for the Irish healthcare system. A conceptual understanding of quality improvement (QI) and patient safety as individual and inter-related fields is required to inform a research agenda. Up to now, research prioritisation exercises in Ireland and internationally have treated these as separate fields. The co-creation of a harmonised Conceptual Framework for Quality Improvement and Patient Safety Research among researchers, knowledge users and patient groups is necessary to outline important topics, research questions and methodologies, applicable and relevant to all areas of healthcare. A framework is defined as ‘a structure, overview, outline, system or plan consisting of various descriptive categories, e.g. concepts, constructs or variables, and the relations between them that are presumed to account for a phenomenon’. ^1^ Therefore we define a QI and patient safety research framework as a set of categories and relations which describe the research process leading to the production of generalisable new knowledge about QI and patient safety. The conceptual framework will enable tailoring of research priorities and methodologies to the needs of service users and staff at unit, organisational and national levels in Ireland. First, a scoping review of the literature is required to examine existing QI and patient safety research frameworks to inform the development of a harmonised conceptual framework for the Irish context. A *scoping* review is the most suitable approach, as it enables the collation, description, and synthesis of information from multiple heterogeneous fields that contribute to the study of QI and patient safety. This paper sets out the background, aims and methodology for the scoping review. The background section defines key concepts, identifies research gaps and discusses relevant health systems developments which inform the review protocol.

## Background

### Quality Improvement

QI is the systemic use of data-guided activities to bring about immediate improvements in healthcare delivery, and is considered a routine part of healthcare. ^2^ In Ireland, the HSE Quality and Patient Safety Competency Navigator outlines the expectation for all healthcare staff to engage in QI activities, to a level commensurate with their role and function in the health service. ^3^ Internationally, QI competency is perceived as essential for supporting quality and safety ^4^ ^–^ ^6^ and United Nations (UN) Sustainable Development Goals (SDGs). ^7^

### Quality Improvement methodologies

QI methodologies include any suite of tools (e.g. process maps, driver diagrams, statistical process control charts) and methods (e.g. model for improvement, lean, six-sigma) which provide a structured scientific approach to making improvements in healthcare. ^8^ According to a scoping review, most published QI initiatives conducted in Ireland (2015–2020) report use of an established QI methodology and achievement of aims. ^9^ The QI initiatives mainly aimed to improve the effectiveness, efficiency, and safety of care processes and outcomes, and few aimed to enhance patient-centredness, value for money, or staff well-being. ^9^ Initiatives rarely aimed to enhance the equity domain of quality, ^9^ an often ‘forgotten goal’ of QI internationally, ^10^ with some recent exceptions from the Irish context. ^11^ Most QI initiatives are unpublished, however the conceptual framework is required to support research questions about all levels of QI work, irrespective of whether the QIs are published and available in the public domain.

### Quality Improvement research

The primary purpose of ‘QI’ is improved care and outcomes near to the time of data collection whereas ‘research’ primarily aims to generate and disseminate new knowledge. ^12^ ^,^ ^13^ The term ‘QI research’ brings these activities together and is defined as ‘the pursuit of *generalizable* new knowledge related to creating and sustaining improvement in health care delivery in real world settings’. ^14^ Additionally, terms such as ‘QI evaluation studies’ and ‘improvement science’ have been use to describe the study of improvement work. ^15^ ^–^ ^18^

The conduct of QI research, like conventional research studies, requires alignment between the study questions and methodology. However, the intervention and the context together are primary parts of the effectiveness of improvement, making traditional high-status methodologies such as randomised control trials an inappropriate fit for the study of improvement. ^19^ General questions important to the field are (a) how can we attain improvement in complex systems?, and (b) how can we improve the way we improve?. ^8^ Dependent on the research question, multiple designs are recommended ^18^ with increasing emphasis on realist syntheses, ^20^ ^,^ ^21^ implementation science, health economic evaluation and behavioural science methods. ^22^

### Quality Improvement research priorities for the Irish healthcare system

QI research is considered to be in its infancy in Ireland and globally. ^8^ There is a need to align designs to important questions, which address a QI knowledge gap, and in particular a QI research priority. General knowledge gaps include the when, why and how of effective adaptation of improvement initiatives to context, and impacts on effectiveness and sustainability. ^23^ In Ireland, even though the National Patient Safety Strategy has established thirteen ‘patient safety improvement priorities’, ^24^ criteria for establishing ‘QI research priorities’ are not yet known. In addition, there is a lack of co-ordination between established improvement priorities and research strategy for the healthcare system. ^24^ ^,^ ^25^ There is also no current guidance on prioritising QI activities across quality domains, settings, and populations, and on selecting appropriate research methodologies and frameworks. Additionally, there is no guidance for identifying which QI initiatives (e.g. small- and/or large-scale) would make a valid and important contribution to *generalisable* new knowledge about creating and sustaining improvement.

A conceptual framework aligning research questions about improvement, with appropriate designs, allied to improvement priorities for the Irish Health System is needed. This framework should integrate fundamentally with patient safety research, given QI’s role in enhancing safe processes and outcomes and the focus of the EQUIPS Research Network.

### Patient safety research

Patient safety is considered an attribute of healthcare systems, ^26^ ^,^ ^27^ and safe systems seek to avoid, prevent and ameliorate adverse outcomes or injuries stemming from care. ^28^ ^,^ ^29^ Patient safety, as a discipline of study, applies ‘safety science methods toward the goal of achieving a trustworthy health system of care’. ^26^ Research is core to enabling the acquisition and application of knowledge to create fail safe and resilient systems. ^26^ ^,^ ^30^ In the early 2000s, the World Health Organisation (WHO) published the Patient Safety Research Cycle which outlined the core research functions of patient safety research. These were 1) measuring harm, 2) understanding the causes of harm, 3) identifying locally affordable solutions, 4) evaluating impact and 5) translating evidence into safer care. ^31^ This is perhaps the most well-known Patient Safety Research Framework, and functions to guide the foci of researchers’ attention worldwide. The 2017 Organisation for Economic Co-operation and Development (OECD) Report ‘The Economics of Patient Safety’ highlighted multiple patient safety solutions which require evaluation and tailoring to local contexts. ^32^ ^,^ ^33^ These and other reports highlight the applied and evaluative nature of patient safety research which aims to lead to the development or adaptation of locally effective, appropriate and affordable solutions. ^32^ ^,^ ^34^

### Patient safety research in Ireland

A 2023 scoping review identified only a modest amount of patient safety research studies conducted in Ireland. ^35^ Most focussed on measuring past harm and safety culture and a minority evaluated patient safety solutions. ^35^ The review called for the availability of patient record data, tailored to distinct aspects of patient harm (e.g. specific care-related injuries, missed diagnoses that lead to harm) to address HSE strategic goals to measure and monitor safety, and establish and evaluate safety improvement initiatives. ^35^

Limitations of the review were a hospital focus, and exclusion of studies which focussed on the safety of one process e.g. medication errors, or patients with a particular medical condition e.g. patients with diabetes. ^35^ For practicality, the scoping review examined safety in the generic sense and studies which addressed many of the 13 HSE patient safety improvement priorities (e.g., falls, pressure ulcers, sepsis etc.) were excluded.

### Patient safety research priorities for the Irish healthcare system

Criteria for establishing patient safety research priorities have evolved over time. In the early 2000s, relevant WHO criteria were ‘the frequency of the safety issue; the magnitude of harm and its distribution within the population; the effect on the efficiency of the health system; the availability, feasibility, and sustainability of solutions; and the urgency or political backing required to tackle the problem’. ^32^ Fifty identified research priorities for developed countries are detailed in WHO reports. ^32^ These were summarised using Donabedian’s structure, process and outcome quality framework to identify research areas that were the results or consequences of care (outcomes), due to resourcing and organisational arrangements (structures) or influenced by the delivery of care (processes). ^36^ It was expected that further knowledge in these substantial knowledge gaps would contribute to improving patient safety. ^32^ Priorities for developed countries like Ireland focussed on the underlying processes and organisational factors that lead to unsafe care, the cost-effectiveness of risk-reducing strategies, and the development of better safety indicators. ^32^

Recently, safety prioritisation exercises have included equity as a key criterion. For example, a 2017 WHO exercise, which consulted global experts, identified research priorities/questions for medication safety research based on the criteria of answerability, effectiveness, innovativeness, implementation, burden reduction and equity. ^37^ In England, an inductive approach to the development of patient safety research priorities for primary care among healthcare staff and patients identified concern for the most vulnerable in society as a key priority. ^38^ The equity emphasis is underpinned by the WHO Global Action Plan for Patient Safety 2021–2030, which has aligned patient safety with United Nation’s Sustainable Development Goals UNSDGs. ^39^ These SDGs reinforce new priority areas in the literature such as the impact of staff well-being on patient safety ^40^ ^,^ ^41^ and the need for environmental sustainability and safety to co-exist, without additional risk to patients. ^42^ Yet, in Ireland, the current criteria for establishing patient safety research priorities have yet to be agreed, as well as the mode for establishing the criteria.

### Irish health system developments

In recent years, new infrastructural developments in the Irish Health System, have linked QI and safety goals, making the current integrated assessment of research priorities timely to inform future policy and practice. For example, Ireland established a National Patient Safety Agency and introduced a new Patient Safety Act, mandating open disclosure. ^43^ Healthcare regions have recently been redefined with the aim of integrating hospital and community care and facilitating the right care at the right time and place. ^44^ Despite Ireland being a relatively late adopter of integrated digital technologies, ^45^ the use of electronic health records, digital solutions and artificial intelligence is expected to increase over the next decade. ^46^ For example, digitisation of data may create greater capacity for QI and patient safety research in Ireland and impact the feasibility of certain research priorities. However, if not carefully implemented, a digital strategy could also contribute to harm and inequities.

Therefore, given the strategic reforms ^47^ and digitisation of healthcare, ^48^ now is an ideal time to ‘design in’ quality and safety into new models of care, and identify relevant quality and safety challenges, and consequent research priorities for the Irish health system. A scoping review is needed to collate all the relevant evidence and inform discussions about harmonised QI and patient safety research priorities for the Irish context.

### Rationale for combining Quality Improvement and Patient Safety Research Frameworks

The US Institute of Medicine have long established patient safety, alongside timeliness, effectiveness, efficiency, equity and patient centredness, as core goals of quality in healthcare. ^49^ Since QI methodologies utilise data to diagnose improvement opportunities, and implement and monitor system performance, there are natural conceptual overlaps between the study of improvement and patient safety research. For example, in healthcare settings, QI methodology (e.g. PDSA) is regularly used to help implement, monitor, and adapt patient safety interventions (e.g. safe surgery checklist) over time with the goal of improving safety. At the same time, using routine and additional data sources, researchers examine implementation and effectiveness outcomes (e.g. compliance with safe surgery checklist and surgical outcomes) to inform decision-making about adoption of policies and practices. For illustration and reflecting the literature above, Table 1, details multiple similarities and differences across QI, QI research and Patient Safety research.

**Table 1.** Similarities and differences in Quality Improvement, Quality Improvement Research, Patient Safety Research.

	QI	QI Research	Patient Safety Research
**Purpose**	Improvement of quality or patient safety in near-to-real in local settings	Creation of generalisable knowledge	Creation of generalisable knowledge
**Guiding Principle**	Pragmatism – what works here?	Pragmatism + scientific rigor	Scientific rigor – what are valid and reliable findings?
**Rationale**	Undesirable variation in one or more quality domains (STEEEP) *, including patient safety	Knowledge gap about how to create and sustain improvement across quality domains (STEEEP) *, including patient safety	Knowledge gap about the scale and causes of patient safety problems and their effective solutions
**Data Sources**	Routine data sources	Routine + research data sources	Routine + research data sources
**Outcomes**	Improved processes and/or staff or patient outcomes	Scientific publication about quality improvement, including patient safety	Scientific publication about patient safety
**Relationship to Patient Safety**	Intersectional - recognises and monitors the influence of other domains on patient safety	Intersectional – recognises and studies the influence of other domains on patient safety and vice versa	Exclusive patient safety focus – aware of other influences but the primary unit of analysis is patient safety (e.g. events, interventions)
**Relationship to QI and QI Research**	Interdependent – culture of QI required to attract and sustain funded QI research opportunities	Interdependent – QI research requires QI initiatives and programmes in place to study them	Independent – can be done without QI initiatives and programmes

* Safety, Timeliness, Effectiveness, Efficiency, Equity, Person-Centredness. ^49^

To reflect these multiple approaches and interdependencies to improving safety in practice, researchers require guidance about selecting evaluation foci and research methodology. For example, a recent Canadian Patient Safety Institute publication, offers guidance on how to combine knowledge translation, quality improvement and implementation science research frameworks, such as the Capability Opportunity and Motivation Model of Behaviour in efforts to enhance patient safety. ^50^ Such a framework could address a recent call for combining QI and PS in clinical education ^51^ and is likely a good example of what could be applied as hybrid research framework, due to its potential to guide the research process and inform knowledge on quality improvement and patient safety. Currently there is no known collated body of literature which profiles stand-alone QI Research Frameworks, Patient Safety Research Frameworks, and hybrid approaches (QI and Patient Safety Research Frameworks) and guides on how these may be best utilised.

## Aims and objectives

The aim of this scoping review is to identify and describe the conceptual frameworks relating to stand-alone QI Research Frameworks, Patient Safety Research Frameworks, and hybrid approaches (QI and Patient Safety Research, QIPS-R, Frameworks) in healthcare. The research questions guiding this review are:

1. What conceptual frameworks exist to guide Quality Improvement and/or Patient Safety Research and how have they been applied in healthcare?
2. How are the concepts of QI and patient safety research defined in Quality Improvement and/or Patient Safety Research Frameworks?
3. What are the reported objectives of Quality Improvement and/or Patient Safety Research frameworks?
4. What domains and sub-domains are addressed in Quality Improvement and/or Patient Safety Research Frameworks?
5. What structure-, process- and outcome-related indicators are used in Quality Improvement and/or Patient Safety Research Frameworks?
6. What criteria for research priorities have been established?
7. What research priorities have been established?

Scoping review findings will be used to inform the development of a meta-framework that will be used to guide QI and patient safety research (QIPS-R) related priority setting, measurement and monitoring, implementation, and knowledge translation activities in Ireland. The actual development of the meta-framework does not form part of the scoping review protocol. The development of the framework will take place in the follow-on phase of this research, as part of EQUIPS Research Network programme of work.

## Methods

The review follows JBI methodological guidance for scoping reviews, ^52^ and will be reported in accordance with the Preferred Reporting Items for Systematic Review and Meta-Analysis (PRISMA) guidelines. The review protocol was structured using JBI guidelines for developing and co-creating scoping review protocols with knowledge users ^53^ ^,^ ^54^ and adaption of the PRISMA extension for Scoping Reviews checklist (PRISMA-ScR). ^55^ All review materials will be available on the Open Science Framework (OSF; https://doi.org/10.17605/OSF.IO/FGWUA).

### Eligibility criteria

The eligibility criteria are informed by a Phenomenon of Interest, Concept, Context approach, as presented in Table 2. We will include all study types across peer reviewed and grey literature. Editorials, book chapters and conference proceedings will be excluded. Since we want to identify frameworks and research priorities applicable to the Irish healthcare context, we will focus on papers from developed countries only, as these will have similar healthcare challenges and research needs. For example, a prior global WHO patient research prioritisation exercise took the approach of identifying priorities for developed, transitional and developing countries. ^31^ Only English language sources will be reviewed due to language limitations of the review team. Similar to other reviews, ^35^ for practicality reasons and to avoid duplication with other research areas, we will exclude papers that focus on the improvement and safety of singular medical processes (e.g. medication safety) and particular patient groups (e.g. patients with diabetes), unless their primary focus is on discussion of research definitions, frameworks, models and/or priorities for QI and/or patient safety research. All healthcare settings will be included. Inclusion thresholds include that papers must address one of the two central scoping review themes: (1) QI and/or patient safety research frameworks and (2) QI and/or patient safety research priorities. Where multiple papers describe the same research frameworks, all papers will be included in the review as supporting references, with the research framework itself only being described once in the scoping review findings.

**Table 2.** Scoping Review Focus.

Phenomenon of Interest	Quality Improvement and/or Patient Safety Research Frameworks used in healthcare
**Concept**	Quality improvement and patient safety research questions, priorities, and methodologies
**Context**	Healthcare settings across the globe

### Information sources and search strategy

A search strategy was developed in consultation with an information specialist (KW) for the selected databases (2000–2025): Ovid MEDLINE, Embase, CINAHL, Scopus, Cochrane Library, Web of Science. Grey literature sources will include international and national government and agency reports, relevant to QI and patient safety research and will be purposively selected from established agencies websites (e.g., World Health Organisation, Agency for Healthcare Research and Quality Patient Safety Network) known to the authors. The search strategy for an example database is presented in Table 3. The full search strategy for each database is available on OSF ( https://doi.org/10.17605/OSF.IO/FGWUA; see Supplementary File 1).

**Table 3.** Database Search Terms (Draft).

("Patient Safety" OR "Healthcare Safety" OR "Safety" OR "Quality Improvement" OR "Improvement Science" Or "Human Factors" Or "Resilience Engineering" Or "Safety I" Or "Safety II" Or "High Reliability" Or "Ergonomics" Or "Socio-technical Systems") AND ("Healthcare") AND ("Research Priorities" OR "Research Framework" OR "Research Methodologies" OR "Research Models" OR "Implementation Science" OR "Behavioural Science" OR "Behaviour Change" OR "Knowledge Translation")

### Data management and study selection

Identified records will be imported to Covidence to manage screening and extraction. Two reviewers will independently assess each of the retrieved records against eligibility criteria. To support inter-rater reliability, seven reviewers (SEM, JP, JMC, EOD, AVC, JAP, CMG) at the study selection stage comprise experienced researchers with PhDs in healthcare quality, human factors and/or health services research or equivalent experience. Titles and abstracts will be screened for relevance, followed by full-text review to examine if eligibility criteria are met. Disagreement among reviewers will be resolved by consulting a third reviewer (SEM plus one other reviewer) to reach consensus. A PRISMA flow diagram will be generated to map out the number of records identified, included and excluded, and the reasons for exclusion.

### Data extraction

Working in teams of two across six reviewers (SEM, JP, EOD, AVC, JAP, CMG), two reviewers (with a balance of healthcare quality/human factors and health services research expertise) will use a piloted, standardised data extraction form, developed in Covidence, to independently extract data relevant to the scoping review aims (see Table 4 for draft template). To support consistency of the process and version control, the extraction template will be finalised and agreed prior to its use it the study. The approach to data extraction will be both deductive and inductive, with some open text extraction options included to enable the collection of concepts and information which may not have been anticipated at the outset ( Table 4). Disagreement among reviewers will be resolved by a third reviewer. To maintain consistency, all consensus decisions will be led be author SEM with at least two other reviewers present.

**Table 4.** Draft Data Extraction Template Headings.

**1. General Information**	
1.1 Title of paper	Free text
1.2 Author(s)	Free text
1.3 Year of Publication	Free text
1.4 Study Digital Object Identifier (DOI)	DOI link
1.5 Country (sl/pl) in which study was conducted	Free text
1.6 Paper type	Theoretical/discursive Literature review Primary research Other
1.7 Quality Improvement and/or Patient Safety Research Definition(s)	Free text
**2. Methods**	
2.1 Study Design	Randomised controlled trial Non-randomised experimental study Cohort study Cross sectional study Case control study Literature review Qualitative research Quantitative research Ethnographic research Quality Improvement research Behavioural Science research Implementation research Implementation effectiveness design Mixed-methods design Prevalence study Case series Case study Diagnostic test accuracy study Clinical prediction rule Health Economic evaluation Text and opinion Participatory Research Delphi Study Other
**3. Theme 1: Quality Improvement (QI) and/or Patient Safety (PS) Research Frameworks**	
3.1 Presentation of a QI and/or PS Research Framework	Yes/No
3.2 Title of Framework	Free text
3.3 Type of QI and/or PS Research Framework	‘Pure Research’ (i.e. to produce generalisable knowledge only) ‘Hybrid Research Plus’ (i.e. to produce generalisable knowledge and other purposes e.g. implementation)
3.4 QI and/or PS Research Framework Focus	Quality Improvement Research Quality Improvement Initiatives Patient Safety Research Implementation Science Behaviour Change Sustainability (i.e. to embed change) Cost and Outcomes Other (free text)
3.5 QI and/or PS Research Framework Aims	Free text (stated aims/goals of framework)
3.6 QI and/or PS Research Framework Domains	Free text (list of domain names or category titles associated with the framework)
3.7 QI and/or PS Research Framework Sub-Domains	Free text (list of framework sub-domains or sub-category titles)
3.8 Framework Recommended Study Designs	Randomised controlled trial Non-randomised experimental study Cohort study Cross-sectional study Case control study Literature review Qualitative research Quantitative research Ethnographic research Quality Improvement research Behavioural science research Implementation research Implementation effectiveness (hybrid) designs Mixed methods designs Prevalence study Case series Case study Diagnostic test accuracy study Clinical prediction rule Health economic evaluation Text and opinion Participatory research Other (free text)
3.9 Recommended Research Methodologies (ie. stated recommended methodologies to measure the framework)	Literature Review Audit Participant Observation Surveys Routine Data Monitoring Run Charts Statistical Process Control Interviews Focus Groups Other (free text)
3.10 If Literature Review Recommend, what type?	Not specified Systematic Review Scoping Review Realist Synthesis Narrative Review Not applicable Other
**4. Theme 2: QI and/or Patient Safety Research Priorities**	
4.1 Were research priorities established?	Yes No Other e.g. research needs/topic recommendations (free text)
4.2 Participants	Population type
4.3 Inclusion criteria	Free text
4.4 Exclusion criteria	Free text
4.5 Total number of participants	Number
4.6 Were criteria for establishing research priorities indicated?	Yes No
4.7 Name and definitions of criteria	Free text
4.8 Number of research priorities established	Number
4.9 Were the research priorities rank ordered?	Yes No Not applicable
4.10 Top 12 research priorities established	Name of each research priority (1-12, if applicable)
4.11 Other proxy priorities i.e. research needs/topic recommendations	Free text research topics Free text interventions Free text methodologies
4.12 Were structural indicators established? (i.e. building blocks of care, e.g. buildings, staff, equipment, policies)	Yes, please specify (free text) No
4.13 Were process indicators established? (i.e. management, organisational, and clinical processes, e.g. use of checklists)	Yes, please specify (free text) No
4.14 Were outcome indicators established? (i.e. outcomes of care, e.g. adverse events)	Yes, please specify (free text) No
4.15 Areas of Care (by Service) for which the priorities were established	All Primary Care Pre-Hospital Care Hospital Care Continuing & Community Care Other (free text)
**5. End Matter**	
5.1 Limitations	Free text
5.2 Study funding sources	Free text
5.3 Possible conflicts of interest for study authors	Free text

### Data analysis and presentation

Tables and graphs will be used to present data which address the study research questions. Findings will demonstrate the breadth of QI and patient safety research frameworks, and will indicate synergies and gaps. Researchers and Knowledge Users will be involved in the analysis and presentation of findings. Scoping review findings will be presented at a dedicated QI and Patient Safety Research symposium at the RCSI University of Medicine & Health Sciences and other fora. Here consultation will take place with stakeholders (i.e. healthcare professionals, healthcare managers, researchers, academics, patient representatives, research funders, and EQUIPS members). Discussion will focus on how the findings may be used to develop Quality Improvement and Patient Safety Research Conceptual Framework and inform the prioritisation of research topics and methodologies within the context of the Irish healthcare system.

### Knowledge user engagement and co-creation

In accordance with the JBI guidance and operating principles of the EQUIPS Research Network, knowledge users (i.e., healthcare staff and patient partners) will co-create all elements of the scoping review. ^54^ The knowledge users are the Lead Knowledge users who volunteered and self-selected to work together with the Researchers in the Strand 2 working group of the EQUIPS Research Network, and those who may join this strand of work as it develops. The first task in the network’s Strand 2 work package primarily concerns establishing relevant research priorities and methodologies, to better facilitate multi-disciplinary and applied research relevant to the HSE patient safety and quality improvement priorities. As such, prior to the development of the scoping review protocol, pre-planning meetings for the conceptualisation of the review took place with knowledge users. ^54^ Then as part of the protocol development, all strand knowledge users were invited to help develop the research questions, search strategy, and review drafts of the protocol manuscript to ensure its helpfulness to their roles. Two such knowledge users in the strand group (TK, JB) supported with these elements. The remainder of the knowledge users could not participate due to constraints on their time from clinical/administrative responsibilities, however, it is anticipated, in accordance with the guidance, they will review results to ‘sense check’ findings and support the development and usability of a QI and Patient Safety Research Conceptual Framework relevant for the Irish context. ^54^ Using their unique experiences, context and perspectives, after the scoping review is published, knowledge users will, alongside the researchers, support dissemination activities, and in ways uniquely relevant to their communities and healthcare settings (e.g. sharing on social media, via podcasts, in specific newsletters, or at journal clubs). ^54^ Where researchers and knowledge users contribute substantially to the work, authorship rights will be applied in accordance with the International Committee of Medial Journal Editors Criteria. ^56^ The overall participatory mechanism for knowledge user engagement with the planned scoping review is the existing schedule of regular online meetings as part of EQUIPS Strand 2 work programme. The scoping review is a standing agenda item at these meetings, which are chaired by the EQUIPS Research Network Manager. The meetings are facilitated with the aim of giving equal voice to researchers and knowledge users. For example, using the meeting forum, knowledge users themselves continually decide how they would like to be included, and contribute to the review. Outputs from the Strand meetings are reported by the Network Manager to the wider network, including its steering committee, whom are responsible for ensuring that strand outputs are used to fulfil the network’s work programme. The actual process of the co-creation of the scoping review will be reported in the final study manuscript using the Guidance for Reporting Involvement of Patients and the Public 2 (GRIPP2) short form checklist, a tool which supports improved reporting of patient and public involvement in research. ^57^

In accordance with JBI guidance, ^54^ there is no requirement to apply for research ethical approval for the scoping review. Knowledge users are being engaged to inform the process of how scoping review is done, and not as research participants. Nevertheless, as is best practice, ethical principles of beneficence and informed consent will be applied to the conduct of the scoping review. Knowledge users, have been informed during online Strand 2 meetings that they are free to share any relevant view and given the volunteer nature of their role in the study, are free to withdraw from participation in the scoping review team for any reason and without incurring any negative consequences from any of the individuals or organisations associated with the research.

## Conclusion

In Ireland, there is a need for greater guidance for researchers involved in studying improvement work and patient safety. The completed review will provide a shared understanding of QI and patient safety research. Based on this understanding the review will enable production of practical tools to assist researchers and improvers. First, the development of a QI and Patient Safety Research Conceptual Framework suitable for the Irish context, that will enable users of the Framework to identify research priorities and methodologies relevant for their areas of work. Secondly, the review will inform a research prioritisation exercise among members of the EQUIPS Research Network, to best inform research funders and policy makers at national level of the key research areas that will make a difference to improving the safety and quality of care in the Irish healthcare system. These dual planned outputs will provide evidenced based priorities and methodologies, and a practical tool to support acceleration of QI and patient safety research in Ireland.

### Dissemination

The paper will be submitted for publication in a peer-reviewed journal, and presented at national and international quality and safety conferences.

### Study status

Between 12 June 2025 and 18 July 2025, the search strategy was developed, piloted, and refined, in consultation with an information specialist. The final search was conducted on 18 July 2025. Title and abstract screening commenced on 21 July 2025. Review of grey literature is ongoing at the time of Version 3 submission (16 April 2026).

### Ethics and consent

Ethical approval and consent were not required.

## Data availability

### Underlying data

No underlying data are associated with this article.

### Extended data

Extended data associated with this article are available on OSF, under the project title “Profiling and Mapping Quality Improvement and Patient Safety Research Frameworks: A Scoping Review to Guide Integration”, https://doi.org/10.17605/OSF.IO/FGWUA (McCarthy, S *et al*. 2025) ^58^

The following extended data are associated with this article:

• PRISMA Scoping Review Checklist_Protocol
• Supplementary File 1_Search strategy

Data are available under the terms of the Creative Commons Attribution 4.0 International Public License (CC BY 4.0).

### Reporting guidelines

An adapted version of PRISMA Scoping Review checklist for the protocol is available on the OSF, under the project title “Profiling and Mapping Quality Improvement and Patient Safety Research Frameworks: A Scoping Review to Guide Integration”, https://doi.org/10.17605/OSF.IO/FGWUA (McCarthy, S *et al*. 2025) ^56^

Data are available under the terms of the Creative Commons Attribution 4.0 International Public License (CC BY 4.0).

## References

[1] Nilsen P Making Sense of Implementation Theories, Models, and Frameworks. In: Albers B Shlonsky A Mildon R , editors. Implementation Science 3.0. 2020; Springer, Cham. 10.1007/978-3-030-03874-8 PMC4406164 25895742

[2] Lynn J Baily MA Bottrell M : The ethics of using quality improvement methods in health care. *Ann Intern Med.* 2007;146(9):666–73. 10.7326/0003-4819-146-9-200705010-00155 17438310

[3] National Quality and Patient Safety : The quality and patient safety competency navigator 2025.V 1.0. Dublin, Ireland.2025. Reference Source

[4] Canadian Patient Safety Institute : *The safety competencies: enhancing patient safety across the health professions.* Alberta: Edmonton;2020. Reference Source

[5] Clinical Excellence Commission: Healthcare safety and quality capabilities : *an occupation-specific set for healthcare workers in NSW health.* Sydney;2021. Reference Source

[6] Welsh Government : Quality and safety framework. 2021. Reference Source

[7] Mensah Abrampah N Syed SB Hirschhorn LR : Quality improvement and emerging global health priorities. *Int J Qual Health Care.* 2018;30(suppl_1):5–9. 10.1093/intqhc/mzy007 29873793 PMC5909628

[8] Reed JE Antonacci G Armstrong N : What is improvement science, and what makes it different? An outline of the field and its frontiers. *Front Health Serv.* 2025;4:1454658. 10.3389/frhs.2024.1454658 40051505 PMC11884242

[9] McCarthy SE Jabakhanji SB Martin J : Reporting standards, outcomes and costs of quality improvement studies in Ireland: a scoping review. *BMJ Open Qual.* 2021;10(3):e001319. 10.1136/bmjoq-2020-001319 34341016 PMC8330587

[10] Ford J Johnson L Blythe J : Evidence brief: what works – leveraging quality improvement to address health and care inequalities.Health Equity Evidence Centre.2025.

[11] Doran E Barron E Healy L : Improving access to epilepsy care for homeless patients in the Dublin Inner City: a collaborative quality improvement project joining hospital and community care. *BMJ Open Qual.* 2021;10(2):e001367. 10.1136/bmjoq-2021-001367 33926992 PMC8094364

[12] Foster J : Differentiating quality improvement and research activities. *Clin Nurse Spec.* 2013;27(1):10–3. 10.1097/NUR.0b013e3182776db5 23222021

[13] Ogrinc G Nelson WA Adams SM : An instrument to differentiate between clinical research and quality improvement. *IRB.* 2013;35(5):1–8. 24350502

[14] Margolis P Provost LP Schoettker PJ : Quality improvement, clinical research, and quality improvement research--opportunities for integration. *Pediatr Clin North Am.* 2009;56(4):831–41. 10.1016/j.pcl.2009.05.008 19660630

[15] Nilsen P Thor J Bender M : Bridging the silos: a comparative analysis of implementation science and improvement science. *Front Health Serv.* 2022;1:817750. 10.3389/frhs.2021.817750 36926490 PMC10012801

[16] Cribb A : Improvement science meets improvement scholarship: reframing research for better healthcare. *Health Care Anal.* 2018;26(2):109–23. 10.1007/s10728-017-0354-6 29270810 PMC5899995

[17] Perla RJ Provost LP Parry GJ : Seven propositions of the science of improvement: exploring foundations. *Qual Manag Health Care.* 2013;22(3):170–86. 10.1097/QMH.0b013e31829a6a15 23807130

[18] Portela MC Pronovost PJ Woodcock T : Republished: how to study improvement interventions: a brief overview of possible study types. *Postgrad Med J.* 2015;91(1076):343–54. 10.1136/postgradmedj-2014-003620rep 26045562 PMC4484358

[19] Øvretveit J : Perspectives: answering questions about quality improvement: suggestions for investigators. *Int J Qual Health Care.* 2017;29(1):137–42. 10.1093/intqhc/mzw136 27837000

[20] Duddy C Wong G : Grand rounds in methodology: when are realist reviews useful, and what does a ‘good’ realist review look like? *BMJ Qual Saf.* 2023;32(3):173–80. 10.1136/bmjqs-2022-015236 36585019

[21] Johnson LL Wong G Kuhn I : A realist review of how, why, for whom and in which contexts quality improvement in healthcare impacts inequalities. *BMJ Qual Saf.* 2025;34(8):537–546. 10.1136/bmjqs-2024-017386 39832840 PMC12322391

[22] Lachman P : *Oxford professional practice handbook of quality improvement in healthcare.* Oxford University Press;2024. 10.1093/med/9780192866387.001.0001 Reference Source

[23] Ovretveit J Dolan-Branton L Marx M : Adapting improvements to context: when, why and how? *Int J Qual Health Care.* 2018;30(suppl_1):20–3. 10.1093/intqhc/mzy013 29878138 PMC5909662

[24] Health Service Executive : *Patient safety strategy 2019-2024.* 2019. Reference Source

[25] Terrés A : *HSE action plan for health research 2019–2029.* 2019. Reference Source

[26] Emanuel L Berwick D Conway J : What exactly is patient safety? *J Med Regul.* 2009;95(1):13–24. 10.30770/2572-1852-95.1.13

[27] Back J Owens D Benneyworth R : The Health Services Safety Investigations Body (HSSIB) and safety management systems: an integrated approach to managing safety in healthcare. *J Patient Saf Risk Manag.* 2024;29(2):79–82. 10.1177/25160435231224583

[28] Grober ED Bohnen JM : Defining medical error. *Can J Surg.* 2005;48(1):39–44. 15757035 3211566 15757035 PMC3211566

[29] Chatzi AV Malliarou M : The need for a nursing specific patient safety definition, a viewpoint paper. *Int J Health Gov.* 2023;28(2):108–116. 10.1108/IJHG-12-2022-0110

[30] Wiig S Aase K Billett S : Defining the boundaries and operational concepts of resilience in the resilience in healthcare research program. *BMC Health Serv Res.* 2020;20(1):330. 10.1186/s12913-020-05224-3 32306981 PMC7168985

[31] World Health Organization : *WHO Patient Safety: WHO patient safety research: better knowledge for safer care.* World Health Organization;2009. Reference Source

[32] Bates DW Larizgoitia I Prasopa-Plaizier N : Global priorities for patient safety research. *BMJ.* 2009;338: b1775. 10.1136/bmj.b1775 19443552

[33] Slawomirski L Auraaen A Klazinga NS : *The economics of patient safety: strengthening a value-based approach to reducing patient harm at national level.* 2017. 10.1787/5a9858cd-en

[34] European Medicines Agency (EMA), Heads of Medicines Agencies (HMA) : Guideline on good pharmacovigilance practices (GVP)—Module XV—Safety communication (Rev 1).EMA/118465/2012 Rev 1.2022. Reference Source

[35] O’Connor P O’Malley R Kaud Y : A scoping review of patient safety research carried out in the Republic of Ireland. *Ir J Med Sci.* 2023;192(1):1–9. 10.1007/s11845-022-02930-1 35122620 PMC8817163

[36] World Alliance for Patient Safety : *Summary of the Evidence on Patient Safety: Implications for Research* Geneva: World Health Organization;2008.

[37] Sheikh A Rudan I Cresswell K : Agreeing on global research priorities for medication safety: an international prioritisation exercise. *J Glob Health.* 2019;9(1):010422. 10.7189/jogh.09.010422 30842883 PMC6393844

[38] Morris RL Stocks SJ Alam R : Identifying primary care patient safety research priorities in the UK: a James Lind Alliance priority setting partnership. *BMJ Open.* 2018;8(2):e020870. 10.1136/bmjopen-2017-020870 29490970 PMC5855454

[39] World Health Organisation : *Global patient safety action plan 2021-2030: towards eliminating avoidable harm in health care.* World Health Organization;2021. Reference Source

[40] Benishek LE Kachalia A Biddison LD : Improving clinician well-being and patient safety through human-centered design. *JAMA.* 2023;329(14):1149–1150. 10.1001/jama.2023.2157 36821124

[41] Ray-Sannerud BN Leyshon S Vallevik VB : Introducing routine measurement of healthcare worker’s well-being as a leading indicator for proactive safety management systems based on resilience engineering. *Procedia Manuf.* 2015;3:319–326. 10.1016/j.promfg.2015.07.163

[42] Lachman P Batalden P Vanhaecht K : A multidimensional quality model: an opportunity for patients, their kin, healthcare providers and professionals to coproduce health [version 3; peer review: 2 approved, 1 approved with reservations]. *F1000Res.* 2020;9:1140. 10.12688/f1000research.26368.3 34158927 PMC8191516

[43] Madden D Tumelty ME : Open disclosure of patient safety incidents: legislative developments in Ireland. *Management.* 2019;179:180.

[44] Committee on the Future of Healthcare : *Slaintecare Report.* 2017. Reference Source

[45] Invent C Page M Winkel R : *2024 digital decade eHealth indicator study.* 2024. Reference Source

[46] Department of Health : *AI for Care, The Artificial Intelligence (AI) Strategy for Healthcare in Ireland (2026-2030).* 2026. Reference Source

[47] Government of Ireland : *Path to Universal Healthcare: Sláintecare & Programme for Government 2025+* Dublin: Government of Ireland;2025.

[48] Department of Health: Digital for care : *a digital health framework for ireland 2024-2030.* 2024. Reference Source

[49] Committee on Quality of Health Care in America : *Crossing the quality chasm: a new health system for the 21st century.* National Academies Press;2001. 25057539

[50] Canadian Patient Safety Institute : *A Guide to Patient Safety Improvement: Integrating Knowledge Translation & Quality Improvement Approaches.* Alberta: Edmonton;2020.

[51] Wong BM Baum KD Headrick LA : Building the bridge to quality: an urgent call to integrate quality improvement and patient safety education with clinical care. *Acad Med.* 2020;95(1):59–68. 10.1097/ACM.0000000000002937 31397709

[52] Peters MD Godfrey C McInerney P : Scoping reviews. *JBI manual for evidence synthesis.* 2020;10. 10.46658/JBIMES-24-09 33038124

[53] Peters MD Godfrey C McInerney P : Best practice guidance and reporting items for the development of scoping review protocols. *JBI Evid Synth.* 2022;20(4):953–68. 10.11124/JBIES-21-00242 35102103

[54] Pollock D Alexander L Munn Z : Moving from consultation to co-creation with knowledge users in scoping reviews: guidance from the JBI scoping review methodology group. *JBI Evid Synth.* 2022;20(4):969–79. 10.11124/JBIES-21-00416 35477565

[55] Tricco AC Lillie E Zarin W : PRISMA extension for Scoping Reviews (PRISMA-ScR): checklist and explanation. *Ann Intern Med.* 2018;169(7):467–73. 10.7326/M18-0850 30178033

[56] Pollock A Campbell P Struthers C : Stakeholder involvement in systematic reviews: a protocol for a systematic review of methods, outcomes and effects. *Res Involv Engagem.* 2017;3:9. 10.1186/s40900-017-0060-4 29062534 PMC5611627

[57] Staniszewska S Brett J Simera I : GRIPP2 reporting checklists: tools to improve reporting of patient and public involvement in research. *BMJ.* 2017;358:j3453. 10.1136/bmj.j3453 28768629 PMC5539518

[58] McCarthy S Poots J McCaffrey J : Profiling and Mapping Quality Improvement and Patient Safety Research Frameworks: A Scoping Review to Guide Integration. *Open Science Framework.* 2025. 10.17605/OSF.IO/FGWUA PMC13044539 41939513

