# Supplementary material for: Profiling and Mapping Quality Improvement and Patient Safety Research Frameworks: Protocol for a Scoping Review to Guide Integration
Source: HRB Open Res. 2026 Apr 28;8:92. Originally published 2025 Aug 18. [Version 3] doi: 10.12688/hrbopenres.14173.3 (PMC13044539; doi:10.12688/hrbopenres.14173.3)
Supplement: Peer review history

# Reviewer report: Reviewer response for version 2

**DOI:** 10.21956/hrbopenres.15774.r54050

**Published:** 2026-04-01

**Authors:** Anthony J Montgomery

**Affiliations:** 1 Northumbria University Newcastle, Newcastle, England, UK

I had the benefit of reading the reviews of three previous reviewers and the response of the authors. It's obvious that the authors have addressed the many technical questions posed by the previous reviewers. Therefore, I will comment on some of the conceptual issues. 

 The basic idea to conduct a scoping review on the research frameworks that guide patient safety and QI research is a worthy one. I am very happy to approve this study, and the following comments should be considered as suggestive, not obligatory. 

 1. It would be helpful for the reader to understand why a scoping review is the best way forward. I assume the scoping review approach reflects the fact that the area is heterogeneous and a little 'scattered', but it would be helpful to comment on this a little. 

 2. Some examples of the research frameworks that already exist would be helpful for the reader. Also, a 'tighter' definition of what is meant by a research framework would be helpful. It sounds a little generic at the moment. For example, what is not a research framework? Will the authors be using inductive/abductive methods to delineate what research frameworks exist?

 3. The research aims are mostly clear. I was just wondering with regard to the first aim, how the authors meant by 'what conceptual frameworks are available' - what is meant by 'available'?

 4. The authors intend to develop a meta-framework, but does this not depend on the quality of the frameworks in existence? For example, if the scoping review finds that the majority of frameworks are poorly described and vague - why try to make a meta-framework? 

 5. The constant use of and/or needs to be clarified a little. For example, what happens if they find many examples of QI that are not directly focused on patient safety issues?  

 I hope my comments have been helpful. As I said, the idea and method are sound, and it will be interesting to see how the authors deal with the heterogeneous nature of the information that they find.

Is the study design appropriate for the research question?

Yes

Is the rationale for, and objectives of, the study clearly described?

Partly

Are sufficient details of the methods provided to allow replication by others?

Yes

Are the datasets clearly presented in a useable and accessible format?

Not applicable

Reviewer Expertise:

Patient safety research, occupational health, burnout, emotional labour, psychological safety.

I confirm that I have read this submission and believe that I have an appropriate level of expertise to confirm that it is of an acceptable scientific standard.

# Response

**Published:** 2026-04-22

**Authors:** John McCaffrey

**Affiliations:** RCSI University of Medicine and Health Sciences, Ireland

**I had the benefit of reading the reviews of three previous reviewers and the response of the authors. It's obvious that the authors have addressed the many technical questions posed by the previous reviewers. Therefore, I will comment on some of the conceptual issues.**

**The basic idea to conduct a scoping review on the research frameworks that guide patient safety and QI research is a worthy one. I am very happy to approve this study, and the following comments should be considered as suggestive, not obligatory.**

**Authors' response**: Thank you very much for the peer review of our scoping review protocol paper. We appreciate the very helpful suggestions around conceptual issues and have responded to these below and incorporated edits in the paper.

**1. It would be helpful for the reader to understand why a scoping review is the best way forward. I assume the scoping review approach reflects the fact that the area is heterogeneous and a little 'scattered', but it would be helpful to comment on this a little**. 

**Authors' response**: Thank you for this helpful suggestion, we have added the below comment to the introductory section:

 "A *scoping *review is the most suitable approach, as it enables the collation, description, and synthesis of information from multiple heterogeneous fields that contribute to the study of QI and patient safety."

**2. Some examples of the research frameworks that already exist would be helpful for the reader. Also, a 'tighter' definition of what is meant by a research framework would be helpful. It sounds a little generic at the moment. For example, what is not a research framework? Will the authors be using inductive/abductive methods to delineate what research frameworks exist?**

**Authors' response**: Thank you for this helpful suggestion. We have now specified what we mean exactly by a QI and patient safety research framework. In the introduction, we have added:

 "A framework is defined as ‘a structure, overview, outline, system or plan consisting of various descriptive categories, e.g. concepts, constructs or variables, and the relations between them that are presumed to account for a phenomenon’. ^1^ Therefore we define a QI and patient safety research framework as a set of categories and relations which describe the research process leading to the production of generalisable new knowledge about QI and patient safety."

 In relation to examples of research frameworks, we are reluctant to put potential findings in the scoping review protocol. However, we have in the existing manuscript, given two examples. To make these more explicit, we highlighted these to the reader a bit more, with the below edits:

 "In the early 2000s, the World Health Organisation (WHO) published the Patient Safety Research Cycle which outlined the core research functions of patient safety research. These were 1) measuring harm, 2) understanding the causes of harm, 3) identifying locally affordable solutions, 4) evaluating impact and 5) translating evidence into safer care. ^31^ This is perhaps the most well-known Patient Safety Research Framework, and functions to guide the foci of researchers’ attention worldwide.

 For example, a recent Canadian Patient Safety Institute publication, offers guidance on how to combine knowledge translation, quality improvement and implementation science research frameworks, such as the Capability Opportunity and Motivation Model of Behaviour in efforts to enhance patient safety. ^50^ Such a framework could address a recent call for combining QI and PS in clinical education ^51^ and is likely a good example of what could be applied as hybrid research framework, due to its potential to guide the research process and inform knowledge on quality improvement and patient safety."

 In response to your helpful suggestion, we have now clarified in the methods section, that we will use both deductive and inductive methods approach to delineate what research frameworks exist. We have added the below to the methods section:

 "The approach to data extraction will be both deductive and inductive, with some open text extraction options included to enable the collection of concepts and information which may not have been anticipated at the outset."

**3. The research aims are mostly clear. I was just wondering with regard to the first aim, how the authors meant by 'what conceptual frameworks are available' - what is meant by 'available'?**

**Authors' response**: Thank you for this query. By available, we mean, what frameworks ‘exist’? To be more clear, we have removed the word ‘available’ the list of objectives. For example, the first objective now reads:

 1. What conceptual frameworks exist to guide Quality Improvement and/or Patient Safety Research and how have they been applied in healthcare?

**4. The authors intend to develop a meta-framework, but does this not depend on the quality of the frameworks in existence? For example, if the scoping review finds that the majority of frameworks are poorly described and vague - why try to make a meta-framework?** 

**Authors' response**: Thank you for this question. This in an important consideration and we cannot pre-empt the findings of the scoping review or what other data sources may be required. This is a good reason why the development of the meta-framework does not form part of the scoping review aims and objectives itself. As indicated in the aims and objectives section, the primary aim of the scoping review itself is to profile existing frameworks. To make this more clear, we have added to the aims and objectives section:

 "The actual development of the meta-framework does not form part of the scoping review protocol. The development of the framework will take place in the follow-on phase of this research, as part of EQUIPS Research Network programme of work."

**5. The constant use of and/or needs to be clarified a little. For example, what happens if they find many examples of QI that are not directly focused on patient safety issues?**  

**Authors' response**: The use of ‘and/or’ is simply to denote that we are profiling a mixed array of frameworks, some may deal with QI or Patient Safety alone, some may deal with both. If we find a QI research framework that is not directly focussed on patient safety issues, that will form part of our findings. It could be that is focussed on other relevant issues that may be indirectly supportive of patient safety research, for example, methodologies for studying how improvement happens. This is explicit in the existing first sentence of the aims and objectives section:

 "The aim of this scoping review is to identify and describe the conceptual frameworks relating to stand-alone QI Research Frameworks, Patient Safety Research Frameworks, and hybrid approaches (QI and Patient Safety Research, QIPS-R, Frameworks) in healthcare."

**I hope my comments have been helpful. As I said, the idea and method are sound, and it will be interesting to see how the authors deal with the heterogeneous nature of the information that they find.**

**Authors' Response**: Thank you

# Reviewer report: Reviewer response for version 2

**DOI:** 10.21956/hrbopenres.15774.r53513

**Published:** 2026-04-01

**Authors:** Mary Lavelle

**Affiliations:** 1 Queen's University Belfast, Belfast, Northern Ireland, UK

Thank you for the opportunity to review this paper. The topic is important, and the scoping review has the potential to offer valuable insights for those working both clinically and from a research perspective within healthcare settings, both in Ireland and beyond.

 It is clear that the authors have made substantial revisions in response to earlier reviewer comments, and these changes have appreciably strengthened both the manuscript and the methodological description.

 At this stage, I have only one remaining query. Will the proposed meta‑framework be presented as part of this review article? If so, it would be helpful to include additional detail on the planned process for co‑creating this framework, including how the development steps will be operationalised.

Is the study design appropriate for the research question?

Yes

Is the rationale for, and objectives of, the study clearly described?

Yes

Are sufficient details of the methods provided to allow replication by others?

Partly

Are the datasets clearly presented in a useable and accessible format?

Not applicable

Reviewer Expertise:

Patient safety and health services research.

I confirm that I have read this submission and believe that I have an appropriate level of expertise to confirm that it is of an acceptable scientific standard, however I have significant reservations, as outlined above.

# Response

**Published:** 2026-04-16

**Authors:** John McCaffrey

**Affiliations:** RCSI University of Medicine and Health Sciences, Ireland

Thank you for your peer review and positive comments on our manuscript. In response to your query, we will not be including the proposed meta-framework as part of the scoping review article. To make this more clear, we have added the below statement at the end of the aims and objectives section:

 The actual development of the meta-framework does not form part of the scoping review protocol. The development of the framework will take place in the follow-on phase of this research, as part of EQUIPS Research Network programme of work

# Reviewer report: Reviewer response for version 2

**DOI:** 10.21956/hrbopenres.15774.r53664

**Published:** 2026-03-04

**Authors:** Kassu Jilcha

**Affiliations:** 1 Addis Ababa University, Addis Ababa, Ethiopia

The study topic is highly relevant to the health sector, and I appreciate the focus on "Profiling and Mapping Quality Improvement and Patient Safety Research Frameworks: Protocol for a Scoping Review to Guide Integration." This work has the potential to serve as a valuable resource for health researchers and healthcare sectors alike. However, there are several areas where the protocol could be strengthened to enhance its scientific rigor and clarity.

**Protocol Structure and Presentation:**

 A well-defined structure is essential for study protocols. The protocol should include a clear Abstract that summarizes the background, objectives, methodology, and anticipated implications, along with appropriate keywords. The Introduction should clearly present the concepts of quality improvement and patient safety, highlighting how these elements can be integrated to enhance patient health outcomes. It should also address the role of health professionals in maintaining quality while providing clinical services and cite recent studies to substantiate these ideas.

**Literature Review and Conceptual Framework:**

 Following the Introduction, a comprehensive Literature Review section should address the specific objectives and research questions while identifying literature gaps. Importantly, this section should culminate in the development of a conceptual framework that currently appears to be missing from the protocol. This framework will be essential for guiding the scoping review methodology.

**Methodology Clarity:**

 The Research Methodology section should comprehensively describe all study areas, including data sources, selection criteria, search strategies, tools to be used for data extraction and quality assessment, and data analysis techniques. This level of detail is crucial for ensuring the protocol's reproducibility and rigor.

**Overall Assessment:**

 The protocol would benefit from more detailed and substantive content throughout, with clear delineation between sections using appropriate headings (Abstract, Introduction, Literature Review, Methodology, and Conclusion). This will help guide readers through the proposed study and facilitate understanding of how the integration of quality improvement and patient safety will be systematically explored.

Is the study design appropriate for the research question?

Yes

Is the rationale for, and objectives of, the study clearly described?

Yes

Are sufficient details of the methods provided to allow replication by others?

Yes

Are the datasets clearly presented in a useable and accessible format?

Yes

Reviewer Expertise:

Occupational safety and health, ergonomics, Quality

I confirm that I have read this submission and believe that I have an appropriate level of expertise to confirm that it is of an acceptable scientific standard, however I have significant reservations, as outlined above.

# Response

**Published:** 2026-04-16

**Authors:** John McCaffrey

**Affiliations:** RCSI University of Medicine and Health Sciences, Ireland

Thank you for your peer review of our scoping review protocol.

 We agree that the rationale for, and objectives of, the study are clearly described; that the study design is appropriate for the research question, and that sufficient details of the methods are provided to allow replication by others. Therefore we agree with your quantitative assessment, but is was mainly unclear how your qualitative assessment matched to this. Nevertheless, we have replied to the qualitative comments, and this supported the revised version.

 Please see below responses to comments provided

- **Protocol Structure and Presentation:**

The manuscript meets with requirements for structuring and describing scoping review protocols (as per JBI guidelines), and we disagree that any further amendments are needed, as the suggestions have already been met. For example, we have covered abstract, title, keywords, introduction, aims/objectives, methods (eligibility criteria, information sources, search strategy, data management, data extraction, data analysis and presentation, knowledge user engagement and co-creation), dissemination. These are all required by JBI standards, and are more reproducible and supportive of researchers than the generic Abstract, Introduction, Literature Review, Methodology, and Conclusion suggested. A heading ‘Literature Review’ is not recommended by JBI, as this would be more substantial than what would be expected for a scoping protocol, particularly when the scoping review itself, once completed, is a form of literature evidence synthesis.

  

- **Literature Review and Conceptual Framework: **

Thank you for your comments on this. We have already included sufficient background literature in the introductory section, as per JBI guidelines for scoping review protocols. We have included definition of terms, why the research is needed, and what the gaps are. In response to your comments, we have added a specific statement on why a scoping review is indicated, to enhance clarity:

 A *scoping *review is the most suitable approach, as it enables the collation, description, and synthesis of information from multiple heterogeneous fields that contribute to the study of QI and patient safety.

 In response to your comments, we have clarified that the development of the conceptual framework does not form part of the scoping review aims and objectives. As per the aims and objectives, we aim to profile existing frameworks, and the development of the framework will form part of the next phase of the research. We have added the below statement:

 The actual development of the meta-framework does not form part of the scoping review protocol. The development of the framework will take place in the follow-on phase of this research, as part of EQUIPS Research Network programme of work.

  

- **Methodological Clarity**: As per your quantitative assessment, and the reviewers 4 and 5, it is obvious the methodological clarity. Your qualitative comments formed a helpful checklist, but these elements – “data sources, selection criteria, search strategies, tools to be used for data extraction and quality assessment, and data analysis techniques” were included in the paper reviewed.

 

- **Overall Assessment:** We agree with the overall quantitative assessment, as per the qualitative comments, we are satisfied that we have structured the protocol headings are per the well-established and internationally accepted and commonly used, JBI guidelines.

# Reviewer report: Reviewer response for version 1

**DOI:** 10.21956/hrbopenres.15586.r51963

**Published:** 2026-01-08

**Authors:** Adam B Sutherland

**Affiliations:** 1 University of Bradford, Bradford, England, UK

Thanks so much for the opportunity to review this paper, which I have read with interest. My interest comes from your position of attempting to tie together safety science with implementation science. I am in agreement with the previous reviewer that this has not been achieved.

 Theoretical Grounding

 The manuscript is weakened by only offering a superficial theoretical exploration of the differences and alignments between improvement science and safety science. One of your review questions (Nr 5) is framed in Donabedian's "Structure-Process-Outcome" structure but there has been no exploration of this as a fundamental principle of quality improvement. Furthermore there has been no attempt to link improvement and safety science principles, which weakens the structure of your proposed analytical approach as it is unclear how you are going to ensure theoretical alignment between the concepts and the future framework you intend to develop.

 End User Involvement

 A core principle of scoping review methodology is the involvement of service users in the creation of the review and its questions. There is only a fleeting reference to service user involvement in the manuscript and I see no service users represented in the author list. I am very strongly on the opinion that where patient safety is involved, patients must be part of the research journey from start to end and this should be reconsidered. 

 Search Strategy

 I've taken some time to look at your search strategies and have concerns that some of the terms you have used may skew your search results. I see behavioural science and implementation science included, human factors and sociotechnical systems but these are all very different concepts. Again,this is related to the lack of theoretical grounding of the proposal. 

 I also wonder about the feasibility of this review. Notwithstanding the theoretical weaknesses I can imagine that the initial search strategies will yield many hundreds of thousands of titles that require screening, and there may be insufficient resource available for this in the methods. 

 Data extraction

 I think a bit more information is needed for the readers to understand what is meant by "QI/PS-R Focus" and "QI/PS-R Definitions" - perhaps this just relates to the theoretical positions taken? How will extractors manage those studies that report BOTH QI and PS factors? I would fully expect that there would be substantial overlap between patient safety and quality improvement. You would need to separate the two concepts and demonstrate how they align (or indeed sit in tension)

Is the study design appropriate for the research question?

Partly

Is the rationale for, and objectives of, the study clearly described?

Yes

Are sufficient details of the methods provided to allow replication by others?

Partly

Are the datasets clearly presented in a useable and accessible format?

Not applicable

Reviewer Expertise:

Human Factors; Patient Safety

I confirm that I have read this submission and believe that I have an appropriate level of expertise to state that I do not consider it to be of an acceptable scientific standard, for reasons outlined above.

# Response

**Published:** 2026-01-28

**Authors:** John McCaffrey

**Affiliations:** RCSI University of Medicine and Health Sciences, Ireland

**Thanks so much for the opportunity to review this paper, which I have read with interest. My interest comes from your position of attempting to tie together safety science with implementation science. I am in agreement with the previous reviewer that this has not been achieved.**

**Response:** Thank you for this observation. Please see reply to reviewer 1. Overall, we have updated the scoping review aims to profiling QI Research Frameworks, Patient Safety Research Frameworks, and Frameworks which may already combine QI and Patient Safety as part of an overall research framework. The paper title has been reorientated to reflect the aims of the scoping review and not a planned application of the findings, i.e. the development of a conceptual framework.

 “Profiling and Mapping Quality Improvement and Patient Safety Research Frameworks: Protocol for a Scoping Review to Guide Integration

**Theoretical Grounding**

**The manuscript is weakened by only offering a superficial theoretical exploration of the differences and alignments between improvement science and safety science**.

**Response:** The paper has incorporated an additional paragraph on the rationale for combining QI and patient safety research frameworks as below. Since part of our data extraction concerns how frameworks define QI and/or patient safety research, the scoping review findings with further detail difference and alignments between these.

 “ **Rationale for combining Quality Improvement and Patient Safety Research Framework**

 The US Institute of Medicine have long established patient safety, alongside timeliness, effectiveness, efficiency, equity and patient centredness, as a core goal of quality in healthcare (IOM, 2001). Since QI methodologies use data for diagnosing improvement opportunities in healthcare systems and for implementing and monitoring improvements in system performance, there are natural conceptual overlaps between the study of improvement and patient safety research. For example, in healthcare settings, QI methodology (e.g. PDSA) is regularly used to help implement, monitor, and adapt patient safety interventions (e.g. safe surgery checklist) over time with the goal of improving safety. At the same time, using routine and additional data sources, researchers examine implementation and effectiveness outcomes (e.g. compliance with safe surgery checklist and surgical outcomes) to inform decision-making about adoption of policies and practices. To reflect the multiple approaches to improving safety in practice, researchers require guidance about selecting evaluation foci and research methodology. For example, a recent Canadian Patient Safety Institute publication, offers guidance on how to combine quality improvement and implementation science research frameworks in efforts to enhance patient safety (CPSI, 2020). Additionally, there was a recent call for QIPS to be combined in clinical education (Wong et al., 2022). Currently there is no known collated body of literature which profiles stand-alone QI Research Frameworks, Patient Safety Research Frameworks, and hybrid approaches (QI and Patient Safety Research Frameworks) and guides on how these may be best utilised.”

**One of your review questions is framed in Donabedian's "Structure-Process-Outcome" structure but there has been no exploration of this as a fundamental principle of quality improvement.**

**Response**: Thank you for this observation. An explanation of Donabedian’s Structure-Process-and Outcome Quality Framework is now provided in the introductory text. Additionally, examples of structures, processes and outcomes are now provided in the study data extraction table (Table 3) in the manuscript, and as identified below (4.12 – 4.14).

 “Fifty identified research priorities for developed countries are detailed in WHO reports ^32^ . These were summarised using Donabedian’s structure, process and outcome quality framework to identify research areas that were the results or consequences of care (outcomes), due to resourcing and organisational arrangements (structures) or influenced by the delivery of care (processes). It was expected that further knowledge in these substantial knowledge gaps would contribute to improving patient safety (WHO, 2008) . Priorities for developed countries like Ireland focused on the underlying processes and organisational factors that lead to unsafe care, the cost-effectiveness of risk-reducing strategies, and the development of better safety indicators ^32”^

 4.12 Were structural indicators established? (i.e. building blocks of care, e.g. buildings, staff, equipment, policies)

 4.13 Were process indicators established? (i.e. management, organisational, and clinical processes, e.g. use of checklists)

 4.14 Were outcome indicators established? (i.e. outcomes of care, e.g. adverse events)

 World Alliance for Patient Safety. Summary of the Evidence on Patient Safety: Implications for Research. Geneva: World Health Organization, 2008

**Furthermore, there has been no attempt to link improvement and safety science principles, which weakens the structure of your proposed analytical approach as it is unclear how you are going to ensure theoretical alignment between the concepts and the future framework you intend to develop.**

**Response**: Thank you for this observation. A table (Table 1) outlining similarities and differences across principles of quality improvement, quality improvement research and patient safety research has been included in the introductory section. Completion of the scoping review, profiling the frameworks, will identify within existing frameworks where synergies across aims, methodology and outcome are, and where there are opportunities for integration.

 “For illustration and reflecting the literature above, Table 1, details multiple similarities and differences across QI, QI research and Patient Safety research. To reflect these multiple approaches and interdependencies in improving safety in practice, researchers require guidance about selecting evaluation foci and research methodology”.

**End User Involvement**

**A core principle of scoping review methodology is the involvement of service users in the creation of the review and its questions. There is only a fleeting reference to service user involvement in the manuscript and I see no service users represented in the author list. I am very strongly on the opinion that where patient safety is involved, patients must be part of the research journey from start to end and this should be reconsidered.**

**Response:** Thank you for this observation. EQUIPS has a small but growing number of patient partners and collaborates regularly with other patient organisations (e.g., Patients for Patient Safety Ireland). All patient partners were invited to participate in scoping review development and we anticipate they will continue to play an active role in further stages of this research. Authors are listed according to the **International Committee of Medical Journal Editors (ICMJE) Recommendations.**

 Please see reply to Reviewer 1 above. More detail has been added on sharing of findings:

 “Scoping review findings will be presented at a dedicated QI and Patient Safety Research symposium at the RCSI University of Medicine & Health Sciences and other fora. Here, consultation will take place with stakeholders (i.e. healthcare professionals, healthcare managers, researchers, academics, patient representatives, research funders, and EQUIPS members). Discussion will focus on how the findings may be used to develop Quality Improvement and Patient Safety Research Conceptual Framework and inform the prioritisation of research topics and methodologies within the context of the Irish healthcare system”.

**Search Strategy**

**I've taken some time to look at your search strategies and have concerns that some of the terms you have used may skew your search results. I see behavioural science and implementation science included, human factors and sociotechnical systems but these are all very different concepts. Again, this is related to the lack of theoretical grounding of the proposal.**

**Response**: Thank you for this observation. We have clarified we are profiling stand-alone and or integrated QI and Patient Safety Frameworks, and have provided more theoretical grounding, I think these address the concerns. Moreover, details of expertise supporting the project are more clearly outlined, and this should instil confidence that papers addressing the theoretical aims of the study will be included.

**I also wonder about the feasibility of this review. Notwithstanding the theoretical weaknesses I can imagine that the initial search strategies will yield many hundreds of thousands of titles that require screening, and there may be insufficient resource available for this in the methods.**

**Response:** We are confident in the feasibility of the review, given our aim to profile *research* frameworks, specifically.

**Data extraction**

**I think a bit more information is needed for the readers to understand what is meant by "QI/PS-R Focus" and "QI/PS-R Definitions" - perhaps this just relates to the theoretical positions taken? How will extractors manage those studies that report BOTH QI and PS factors? I would fully expect that there would be substantial overlap between patient safety and quality improvement. You would need to separate the two concepts and demonstrate how they align (or indeed sit in tension)**

**Response: **Thank you for this observation. I think this had been addressed by our clarification of aims. Please see reply to reviewer 1 above and below

 We have clarified our overall aim is identify stand-alone QI Research Frameworks, Patient Safety Research Frameworks, and hybrid approaches (QI and Patient Safety Research, QIPS-R, Frameworks), and not necessarily hybrid approaches only, which are in their infancy. Additionally, we have clarified that the results of the scoping review will be used to develop a meta-framework, the methodology for which is beyond the parameters of the scoping review protocol.

 “The aim of this scoping review is to identify and describe the conceptual frameworks relating to stand-alone QI Research Frameworks, Patient Safety Research Frameworks, and hybrid approaches (QI and Patient Safety Research, QIPS-R, Frameworks) in healthcare. The research questions guiding this review are:

 1. What conceptual frameworks are available for Quality Improvement and/or Patient Safety Research and how have they been applied in healthcare?

 2. How are the concepts of QI and patient safety research defined in available Quality Improvement and/or Patient Safety Research Frameworks?

 3. What are the reported objectives of available Quality Improvement and/or Patient Safety Research frameworks?

 4. What domains and sub-domains are addressed in available Quality Improvement and/or Patient Safety Research Frameworks?

 5. What structure-, process- and outcome- related indicators are used in available Quality Improvement and/or Patient Safety Research Frameworks?

 6. What criteria for research priorities have been established?

 7. What research priorities have been established?

 Scoping review findings will be used to inform the development of a meta-framework that will be used to guide QI and patient safety research related priority setting, measurement and monitoring, implementation, and knowledge translation activities in Ireland.”

# Reviewer report: Reviewer response for version 1

**DOI:** 10.21956/hrbopenres.15586.r50211

**Published:** 2025-11-22

**Authors:** Olalekan John Okesanya

**Affiliations:** 1 Neuropsychiatric Hospital, Aro, Nigeria

The paper suffers from multiple deficiencies, as mentioned below, which diminish its academic value.

 The paper aims to integrate quality improvement (QI) and patient safety (PS), though the scarcity of a clear theoretical model linking these concepts hinders the strength of the proposed meta-framework with insufficient explanation of their connection. Moreover, while “knowledge user engagement” is emphasised, the protocol offers minimal discussion on the participatory mechanisms involved. The omission of concrete example regarding decision-making structures leaves the co-creation assertion somewhat unsupported. Also, the authors state ethical approval is unnecessary yet moral considerations in stakeholder engagement and interpretation are overlooked parting the ethical structure incomplete. Equally, although dual independent reviewers are noted, key details about their training and calibration are left out this failure raises concerns regarding consistency in inclusion decisions and inter rater reliability. In addition, the draft extraction template is presented, though it is vague in relation to coding schemes and variable definitions, making it difficult to understand the way multi-domain data will be categorized. Furthermore, the review’s findings aim to contribute to a conceptual framework, but the process for developing this framework remains unaddressed, making it unclear whether it should be inductively derived or theoretically imposed. Meanwhile, covidence is mentioned, lacking elaboration on how data validation, version control and reviewer calibration will be managed raising concerns about internal consistency and data integrity. Then, the adherence to JBI and PRISMA-ScR guidelines is outlined though procedural details on how these frameworks will be executed remain inadequately clarified. The shortcoming of explicit inclusion thresholds and screening examples impacts the review’s reproducibility, rendering it questionable. Subsequently, the research plans to retrieve multiple frameworks addressing similar concepts, yet a method for reconciling duplicates as well as overlaps is absent which could result in data redundancy, distorting the synthesis. Finally, the Irish healthcare system is described in broad terms, without addressing system-specific factors that could influence the research, limiting its ability to provide contextually relevant recommendations.

Is the study design appropriate for the research question?

No

Is the rationale for, and objectives of, the study clearly described?

No

Are sufficient details of the methods provided to allow replication by others?

No

Are the datasets clearly presented in a useable and accessible format?

No

Reviewer Expertise:

IoT, Health care, AI and Cloud

I confirm that I have read this submission and believe that I have an appropriate level of expertise to state that I do not consider it to be of an acceptable scientific standard, for reasons outlined above.

# Response

**Published:** 2026-01-28

**Authors:** John McCaffrey

**Affiliations:** RCSI University of Medicine and Health Sciences, Ireland

Thank you for time and expertise in providing the peer review. We have reflected on each of the comments and provide the below clarifications and revisions, which have strengthened and clarified the academic value of our planned study.

**Theoretical Rationale for Study Research Questions**

1. **The paper aims to integrate quality improvement (QI) and patient safety (PS), though the scarcity of a clear theoretical model linking these concepts hinders the strength of the proposed meta-framework with insufficient explanation of their connection.**

**Response:** We have revised the study background to make clear the conceptual overlap between QI Research and Patient Safety Research and have clarified the study aims and objectives. We are aware that QI Research Frameworks exist in the literature, as do Patient Safety Research Frameworks. These are valid and are required to guide researchers in their fields. We have added a definition of a framework to the introductory paragraph:

 A framework is considered ‘a structure, overview, outline, system or plan consisting of various descriptive categories, e.g. concepts, constructs or variables, and the relations between them that are presumed to account for a phenomenon’ ^1^

 Nilsen, P. Making Sense of Implementation Theories, Models, and Frameworks. In: Albers, B., Shlonsky, A., Mildon, R. (eds) Implementation Science 3.0. 2020. Springer, Cham. https://doi.org/10.1007/978-3-030-03874-8.

 We are also aware that both QI research and Patient Safety research address overlapping goals (i.e. contributing to knowledge to enhance safety), and while we are aware of some recent frameworks that guide both QI and Patient Safety Research (e.g. Canadian Patient Safety Institute (2020) A Guide to Patient Safety Improvement: Integrating Knowledge Translation & Quality Improvement Approaches), there is no systematic collation of such frameworks available from the literature that we could identify. The availability of existing frameworks like this strengthens our proposal to develop a meta-framework, which could be used partially or in-full dependent on the needs of end-users such as those within our diverse network. For example, researchers may take guidance from a full meta-framework or elements of it, dependent on their context or the aims of their study. For health professionals, there has been a call to integrate quality improvement and patient safety research in clinical education ( Wong et al., 2020) which a meta-framework could support . In essence, individual QI and Patient Safety Research Frameworks are still required, but since they are part of the knowledge generation and translation continuum that aims to improve safety, a meta-research framework is required to reflect and understand the co-occurring approaches to improving patient safety in practice. As such, the primary aim of this scoping review is to profile stand-alone QI Research Frameworks, Patient Safety Research Frameworks, and hybrid approaches (QI and Patient Safety Research Frameworks). Thereafter, at the end of the scoping review study, we will use this understanding to inform the development of a meta-framework. We appreciate this was not sufficiently clear in the original submission. We have added the below paragraph on the rationale for combining the QI and patient safety research frameworks, and have clarified our review aims and objectives.

 “ **Rationale for combining Quality Improvement and Patient Safety Research Framework**

 The US Institute of Medicine have long established patient safety, alongside timeliness, effectiveness, efficiency, equity and patient centredness, as a core goal of quality in healthcare (IOM, 2001). Since QI methodologies use data for diagnosing improvement opportunities in healthcare systems and for implementing and monitoring improvements in system performance, there are natural conceptual overlaps between the study of improvement and patient safety research. For example, in healthcare settings, QI methodology (e.g. PDSA) is regularly used to help implement, monitor, and adapt patient safety interventions (e.g. safe surgery checklist) over time with the goal of improving safety. At the same time, using routine and additional data sources, researchers examine implementation and effectiveness outcomes (e.g. compliance with safe surgery checklist and surgical outcomes) to inform decision-making about adoption of policies and practices. To reflect the multiple approaches to improving safety in practice, researchers require guidance about selecting evaluation foci and research methodology. For example, a recent Canadian Patient Safety Institute publication, offers guidance on how to combine quality improvement and implementation science research frameworks in efforts to enhance patient safety (CPSI, 2020). Additionally, there was a recent call for QIPS to be combined in clinical education (Wong et al., 2022). Currently there is no known collated body of literature which profiles stand-alone QI Research Frameworks, Patient Safety Research Frameworks, and hybrid approaches (QI and Patient Safety Research Frameworks) and guides on how these may be best utilised.”

 Institute of Medicine, Committee on Quality of Health Care in America. Crossing the quality chasm: a new health system for the 21st century. National Academies Press; 2001.

 Canadian Patient Safety Institute. A Guide to Patient Safety Improvement: Integrating Knowledge Translation & Quality Improvement Approaches. Edmonton, Alberta; 2020

 We have clarified our overall aim is identify stand-alone QI Research Frameworks, Patient Safety Research Frameworks, and hybrid approaches (QI and Patient Safety Research, QIPS-R, Frameworks), and not necessarily hybrid approaches only, which are in their infancy. Additionally, we have clarified that the results of the scoping review will be used to develop a meta-framework, the methodology for which is beyond the parameters of the scoping review protocol.

 “The aim of this scoping review is to identify and describe the conceptual frameworks relating to stand-alone QI Research Frameworks, Patient Safety Research Frameworks, and hybrid approaches (QI and Patient Safety Research, QIPS-R, Frameworks) in healthcare. The research questions guiding this review are:

 1. What conceptual frameworks are available for Quality Improvement and/or Patient Safety Research and how have they been applied in healthcare?

 2. How are the concepts of QI and patient safety research defined in available Quality Improvement and/or Patient Safety Research Frameworks?

 3. What are the reported objectives of available Quality Improvement and/or Patient Safety Research frameworks?

 4. What domains and sub-domains are addressed in available Quality Improvement and/or Patient Safety Research Frameworks?

 5. What structure-, process- and outcome- related indicators are used in available Quality Improvement and/or Patient Safety Research Frameworks?

 6. What criteria for research priorities have been established?

 7. What research priorities have been established?

 Scoping review findings will be used to inform the development of a meta-framework that will be used to guide QI and patient safety research related priority setting, measurement and monitoring, implementation, and knowledge translation activities in Ireland.”

**Participatory Mechanisms for Knowledge User Engagement**

1. **Moreover, while “knowledge user engagement” is emphasised, the protocol offers minimal discussion on the participatory mechanisms involved. The omission of concrete example regarding decision-making structures leaves the co-creation assertion somewhat unsupported.**

**Response:** Thank you for this observation. We have added the below paragraph to the section on knowledge-user engagement and co-creation.

 “The overall participatory mechanism for knowledge user engagement with the planned scoping review is the existing schedule of regular online meetings as part of EQUIPS Strand 2 work programme. The scoping review is a standing agenda item at these meetings, which are chaired by the EQUIPS Research Network Manager. The meetings are facilitated with the aim of giving equal voice to researchers and knowledge users. For example, using the meeting forum, knowledge users themselves continually decide how they would like to be included, and contribute to the review. Outputs from the Strand meetings are reported by the Network Manager to the wider network, including its steering committee, whom are responsible for ensuring that strand outputs are used to fulfil the network’s work programme”.

**Ethical Considerations**

1. **Also, the authors state ethical approval is unnecessary yet moral considerations in stakeholder engagement and interpretation are overlooked parting the ethical structure incomplete**

**Response:** Thank you for this observation, we have expanded the ethical considerations and have included the below paragraph:

 “In accordance with JBI guidance ^50^, there is no requirement to apply for research ethical approval for the scoping review. Knowledge users are being engaged to inform the process of how scoping review is done, and not as research participants. Nevertheless, as is best practice, ethical principles of beneficence and informed consent will be applied to the conduct of the scoping review. Knowledge users, have been informed during online Strand 2 meetings that they are free to share any relevant view and given the volunteer nature of their role in the study, are free to withdraw from participation in the scoping review team for any reason and without incurring any negative consequences from any of the individuals or organisations associated with the research.”

**Methodology**

**Reviewers **

1. **Equally, although dual independent reviewers are noted, key details about their training and calibration are left out this failure raises concerns regarding consistency in inclusion decisions and inter rater reliability. **

**Response**: Thank you for this observation. We have added the below to the methodology section.

 “Two reviewers will independently assess each of the retrieved records against eligibility criteria. To support inter-rater reliability, seven reviewers (SEM, JP, JMC, EOD, AVC, JAP, CMG) at the study selection stage comprise experienced researchers with PhDs in healthcare quality, human factors and/or health services research or equivalent experience. Titles and abstracts will be screened for relevance, followed by full-text review to examine if eligibility criteria are met. Disagreement among reviewers will be resolved by consulting a third reviewer (SEM plus one other reviewer) to reach consensus.”

 Additionally:

 “Working in teams of two across six reviewers (SEM, JP, EOD, AVC, JAP, CMG), two reviewers (with a balance of healthcare quality/human factors and health services research expertise) will use a piloted, standardised data extraction form, developed in Covidence, to independently extract data relevant to the scoping review aims (see Table 4 for draft template).”

**Data Extraction Template**

1. **In addition, the draft extraction template is presented, though it is vague in relation to coding schemes and variable definitions, making it difficult to understand the way multi-domain data will be categorized. **

**Response:** Thank you for this observation. An updated draft extraction template is now presented, with better clarity and includes variable definitions where relevant. Please see Table 4 in the revised manuscript.

**Review Findings**

1. **Furthermore, the review’s findings aim to contribute to a conceptual framework, but the process for developing this framework remains unaddressed, making it unclear whether it should be inductively derived or theoretically imposed**.

**Response:** Thank you for this observation. We have now clarified that our aim is to identify and describe the conceptual frameworks relating to stand-alone QI Research Frameworks, Patient Safety Research Frameworks, and hybrid approaches (QI and Patient Safety Research, QIPS-R, Frameworks) in healthcare. While we indicate that the results will be used to contribute to a conceptual framework, describing the process for this is beyond the scope of the review protocol paper. However, we have added to the methodology section that the scoping review findings will be presented to stakeholders at a QI and Patient Safety Research Symposium and other for a. Here consultation about how the findings could be used to inform a framework for Irish context will take place. See paragraph below:

 “Scoping review findings will be presented at a dedicated QI and Patient Safety Research symposium at the RCSI University of Medicine & Health Sciences and other fora. Here consultation will take place with stakeholders (i.e. healthcare professionals, healthcare managers, researchers, academics, patient representatives, research funders, and EQUIPS members). Discussion will focus on how the findings may be used to develop a QI and Patient Safety Research Conceptual Framework and inform the prioritisation of research topics and methodologies within the context of the Irish healthcare system.”

 In addition, we have updated the title of the scoping review protocol to make this more clear.

 “Profiling and Mapping Quality Improvement and Patient Safety Research Frameworks: Protocol for a Scoping Review to Guide Integration”

**Covidence**

1. **Meanwhile,** **C** **ovidence is mentioned, lacking elaboration on how data validation, version control and reviewer calibration will be managed raising concerns about internal consistency and data integrity. **

**Response:** Thank you for this observation. We have updated the data extraction section as below to address these concerns.

 “To support consistency of the process and version control, the extraction template will be finalised and agreed prior to its use it the study. Disagreement among reviewers will be resolved by a third reviewer. To maintain consistency, all consensus decisions will be led be author SEM with at least two other reviewers present.”

**Adherence to JBI and PRISMA-ScR**

1. **Then, the adherence to JBI and PRISMA-ScR guidelines is outlined though procedural details on how these frameworks will be executed remain inadequately clarified. The shortcoming of explicit inclusion thresholds and screening examples impacts the review’s reproducibility, rendering it questionable. Subsequently, the research plans to retrieve multiple frameworks addressing similar concepts, yet a method for reconciling duplicates as well as overlaps is absent which could result in data redundancy, distorting the synthesis. **

**Response:** Thank you for this observation. We believe the inclusion of a detailed data extraction template addresses the concern about application of JBI and PRISMA-ScR guidelines. To address the issue of inclusion thresholds we have added the below statement under the study eligibility criteria

 “Inclusion thresholds include that papers must address one of the two central scoping review themes: (1) QI and/or patient safety research frameworks and (2) QI and/or patient safety research priorities”.

 To address the issue of duplicates, papers which address the same framework will be included as a supporting reference, but the framework will only be described once in the scoping review report.

 “Where multiple papers describe the same research frameworks, all papers will be included in the review as supporting references, with the research framework itself only being described once in the scoping review findings”.

**Contextual Relevance – Irish Healthcare System**

1. **Finally, the Irish healthcare system is described in broad terms, without addressing system-specific factors that could influence the research, limiting its ability to provide contextually relevant recommendations.**

**Response:** Thank you for this observation which is well founded. As we have now revised the study aim (to produce a scoping review) over production of a conceptual framework, this critique means our scoping review findings ought to be contextualised with the context of the Irish healthcare system. Planned consultation with stakeholders at the QI and patient safety research symposium will enable this. We have updated the methodology as below:

 “Scoping review findings will be presented at a dedicated QI and Patient Safety Research symposium at the RCSI University of Medicine & Health Sciences and other fora. Here consultation will take place with stakeholders (i.e healthcare. professionals, healthcare managers, researchers, academics, patient representatives, research funders, and EQUIPS members) on how the findings may be used to develop a research conceptual framework and inform research prioritisation exercises within the context of the Irish healthcare system”.
